# Combined effect of seasons and life history in an anuran strengthens the response and relationship between their physiology and gut microbiota

**DOI:** 10.1038/s41598-024-60105-7

**Published:** 2024-05-02

**Authors:** Jun-Kyu Park, Yuno Do

**Affiliations:** Department of Biological Sciences, Kongju National University, (32588) Room 204, 56, Kongjudaehak-Ro, Kongju-si, Chungcheongnam-do 32588 Republic of Korea

**Keywords:** Amphibian, Ectotherms, Host physiology, Gut microbiota, Phenology, Ecophysiology, Stable isotope analysis, Microbial ecology, Community ecology, Immunology, Zoology

## Abstract

Gut microbiota impact host physiology, though simultaneous investigations in ectothermic vertebrates are rare. Particularly, amphibians may exhibit more complex interactions between host physiology and the effects of gut microbiota due to the combination of seasonal changes and complex life histories. In this study, we assessed the relationships among food resources, gut bacterial communities, and host physiology in frogs (*Phelophylax nigromaculatus*), taking into account seasonal and life history variations. We found that food sources were not correlated with physiological parameters but had some relationships with the gut bacterial community. Variations in gut bacterial community and host physiology were influenced by the combined effects of seasonal differences and life history, though mostly driven by seasonal differences. An increase in Firmicutes was associated with higher fat content, reflecting potential fat storage in frogs during the non-breeding season. The increase in Bacteroidetes resulted in lower fat content in adult frogs and decreased immunity in juvenile frogs during the breeding season, demonstrating a direct link. Our results suggest that the gut microbiome may act as a link between food conditions and physiological status, and that the combined effect of seasons and life history could reinforce the relationship between gut microbiota and physiological status in ectothermic animals. While food sources may influence the gut microbiota of ectotherms, we contend that temperature-correlated seasonal variation, which predominately influences most ectotherms, is a significant factor.

## Introduction

The direct impact of gut microbiota on animal physiology is recognized through symbiosis and co-evolution with the host^1,2^. Endothermic vertebrates, including humans, have been the main focus of gut microbiota research. Compared to ectothermic vertebrates, studies on their physiological processes or response mechanisms are more developed^3,4^. There has been a recent increase in studies on ectothermic animals' gut microbiota, but still, this area needs more investigation due to the relative lack of research compared to endothermic creatures. The knowledge gap is pronounced and understanding gut microbiota in ectothermic animals can be a complex task. Ectothermic animals' body temperature is determined by external factors, and that affect the gut microbiota^5,6^. Most research involving these creatures has focused on comparing microbial communities in relation to health status, field investigation, and exposure to environmental factors^7–9^. However, our understanding of the direct relationship between an ectothermic host's physiological state and its gut microbiota remains incomplete. Numerous studies have indicated potential impacts of gut microbiota changes on aspects such as immunity, digestive efficiency, and energy metabolism. Further research is necessary to quantify these interactions and elucidate the mechanisms of response. Through such studies, we can achieve a more detailed insight into the response of animal health to these complex interactions, thus advancing our knowledge of the field.

Frogs may undergo more drastic environmental changes that can affect their gut microbiota and physiology compared to other vertebrates. As most frog species alternate between terrestrial and aquatic habitats throughout their life cycles, they experience a variety of environmental combinations^10,11^. Specifically, the physiological changes during metamorphosis from tadpoles to adults and the seasonal patterns of reproduction in temperate regions represent extreme environmental shifts. One of the most significant changes during metamorphosis is the transition from an herbivorous to a carnivorous diet, which accompanies substantial modifications in visceral organs^12^. These changes in gastric or intestinal morphology and diet during metamorphosis profoundly alter the diversity and community composition of the gut microbiota^13^. The physiological effects of life history are important because they can affect not only a single life history, but also later life stages^14,15^.

As a result, we can expect variations between juvenile frogs, who are in a post-metamorphosis growth phase, and adults, whose growth is complete. These differences in gut microbiota homeostasis and physiological stability may stem from the complexities of amphibian life history and physical growth factors, such as body surface area and weight. It is also important to note that their biology, including food source, reproduction, physiology, and metabolism, are subject to seasonal changes, especially temperature variations in temperate regions^16^. Winter hibernation halts feeding activities, modifies physiology, and significantly reduces metabolic rate, affecting processes such as digestion and glucose metabolism^17,18^. Additionally, hormonal levels and energy metabolism may shift during the breeding season post-hibernation^19,20^ and in non-breeding seasons in preparation for hibernation^21,22^. Thus, the varied experiences of life history and seasonal changes can trigger more complex responses when combined as opposed to when they occur individually.

In this study, we aimed to ascertain whether a combination of life history and seasonal changes affects the physiological state and relationship of black-spotted pond frogs (*Phelophylax nigromaculatus*), encompassing factors such as dietary composition, gut bacterial community, body composition, and innate immunity. Previous research revealed that shifts in insect fauna, encompassed by seasonal variations, lead to alterations in the food source, as evidenced by gut contents, thereby influencing the gut microbiota^23^. However, these changes in food sources might also be integrated as part of the overall seasonal transformation.

Furthermore, although changes in the gut microbiota may vary between host species and in the intensity of their response, these alterations are not merely short-term phenomena. Thus, using techniques like stable isotope analysis, which reflects food sources over longer terms (weeks or months), and aligning the response timescales of both the microbiota and the food source can enhance the accuracy of our inferences^24^. Carbon stable isotope ratios enable the identification of the origin of consumed food sources, while nitrogen stable isotope ratios offer insights into an individual's trophic level^25,26^. By combining these two isotope ratios, one can estimate both food resource availability and prey contribution^27,28^.

To evaluate the frogs' physiological state, we analyzed body composition, an indicator of energy storage and metabolism^29,30^, and innate immunity, which can signify resilience against diseases^31,32^. Food sources can directly influence amphibian physiology, such as body composition and immunity^29,33^, and potentially alter physiology through interactions with gut microbiota. Notably, fat serves as a primary energy source for amphibians, from reproduction to successful hibernation^17,34^, making the understanding of its interaction with gut microbiota, particularly in a seasonal context, essential. Additionally, immunity plays a crucial role in population sustainability and presents significant challenges for amphibians, with disease infection being a primary factor in population decline^35^. To enhance the conservation and ecological understanding of amphibians, it is crucial to explore the dynamics of immune responses across seasonal and life cycle changes, particularly during periods of instability. We anticipated that adult and juvenile frogs would exhibit distinct physiological variation (immunity and fat ratio) by seasonal difference, with even more pronounced differences of host physiology and gut microbiota observed in juvenile frogs. Conversely, adaptation to life history in adult frogs may be more stable compared to juvenile frogs, even though energy-intensive breeding behaviors like spawning and advertisement calling during the breeding season represent significant energy expenditure throughout their life history^20,36^. Though identifying and accurately interpreting physiological responses to large and intricate factors, a blend of life history and seasonal responses, can be an immensely challenging task, it is a crucial step toward understanding these phenomena in real-world contexts. Ultimately, we elucidated a relationship between food source, gut bacterial community, and physiology, furthering our understanding of these interactions in ectothermic animals.

## Results

### Comparison of stable isotope ratio

**Figure 1:**
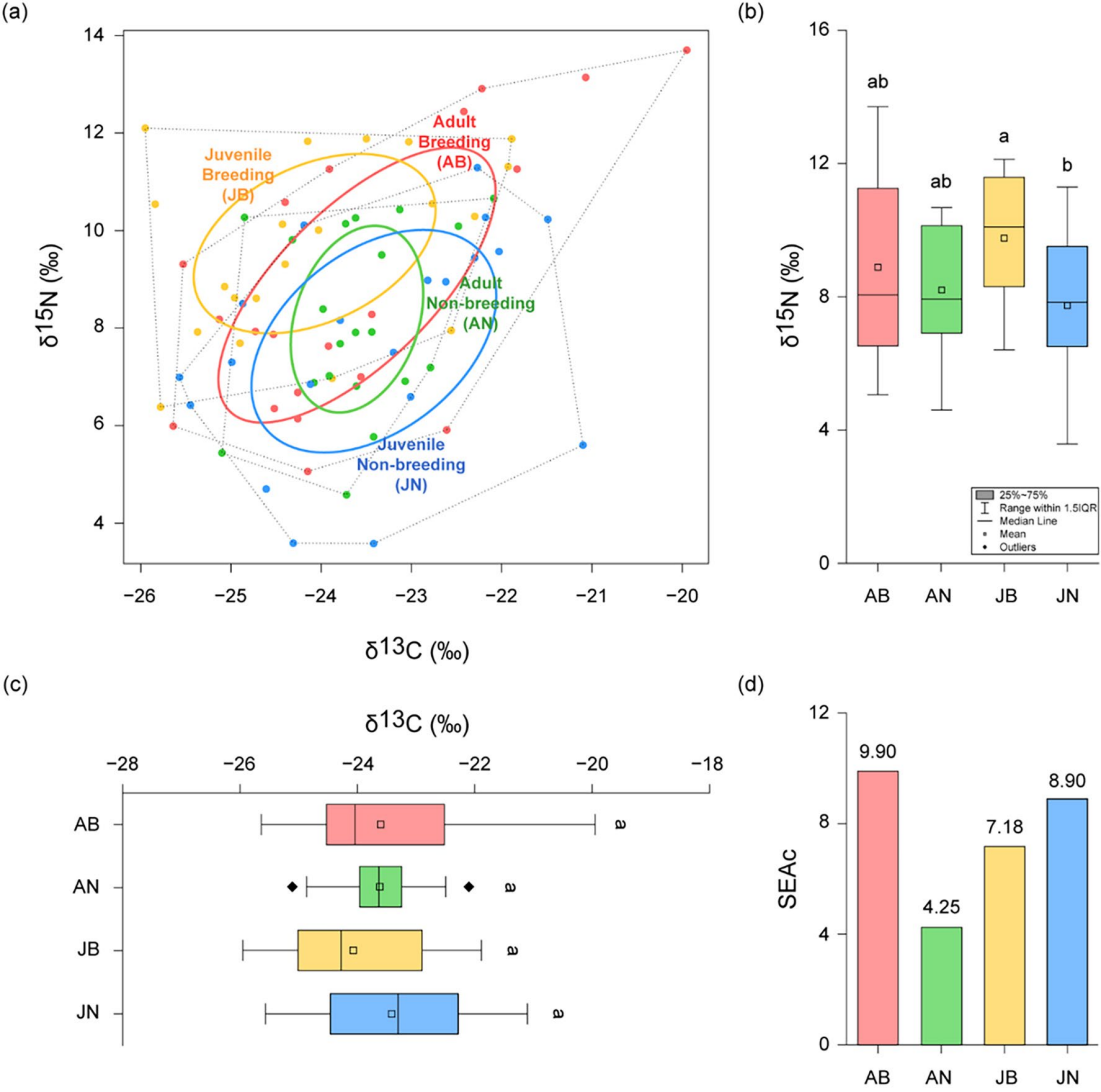
Food resource availability and δ^13^C and δ^15^N values of adult breeding (AB), adult non-breeding (AN), juvenile breeding (JB), and juvenile non-breeding frogs (JN). (**a**) Standard ellipse areas and convex hull, which estimate food resource availability, among the four frog groups, (**b**) Comparison of δ^15^N values among the four groups, (**c**) Comparison of δ^13^C values among the four groups, (**d**) Comparison of food resource availability (standard ellipse area) among the four groups. Significant differences (*p* < 0.05) were determined using a Dunn’s post hoc test following a Kruskal–Wallis test, and are indicated by lowercase letters in panels (**b**) and (**c**).

We compared the food sources among adult breeding (AB), adult non-breeding (AN), juvenile breeding (JB), and juvenile non-breeding (JN) frogs. There was no significant difference (H = 3.26, *p* = 0.35) in the carbon stable isotope ratio (δ^13^C) was observed, whereas the nitrogen stable isotope ratio (δ^15^N) showed a slight difference (H = 8.57, *p* = 0.04) (Fig. 1a–c). The difference in δ^15^N between adult and juvenile frogs in both the breeding and non-breeding seasons was not observed (Dunn’s post hoc, *p* > 0.05). In adult frogs, the δ^15^N values did not differ significantly by season (Dunn’s post hoc, *p* > 0.05), whereas juvenile frogs had higher δ^15^N ratios during the breeding season compared to the non-breeding season (Dunn’s post hoc test, *p* < 0.05). The sample size-corrected standard ellipse area (SEAc), which indicates food resource availability estimated by using δ^13^C and δ^15^N, was highest in adult frogs during the breeding season (SEAc = 9.90) and lowest during the non-breeding season (SEAc = 4.25). In juvenile frogs, a higher SEAc was observed in the non-breeding season (SEAc = 8.90) than in the breeding season (SEAc = 7.18), which was in contrast to the pattern observed in adult frogs (Fig. 1d).

### Diversity of gut bacterial community

**Figure 2:**
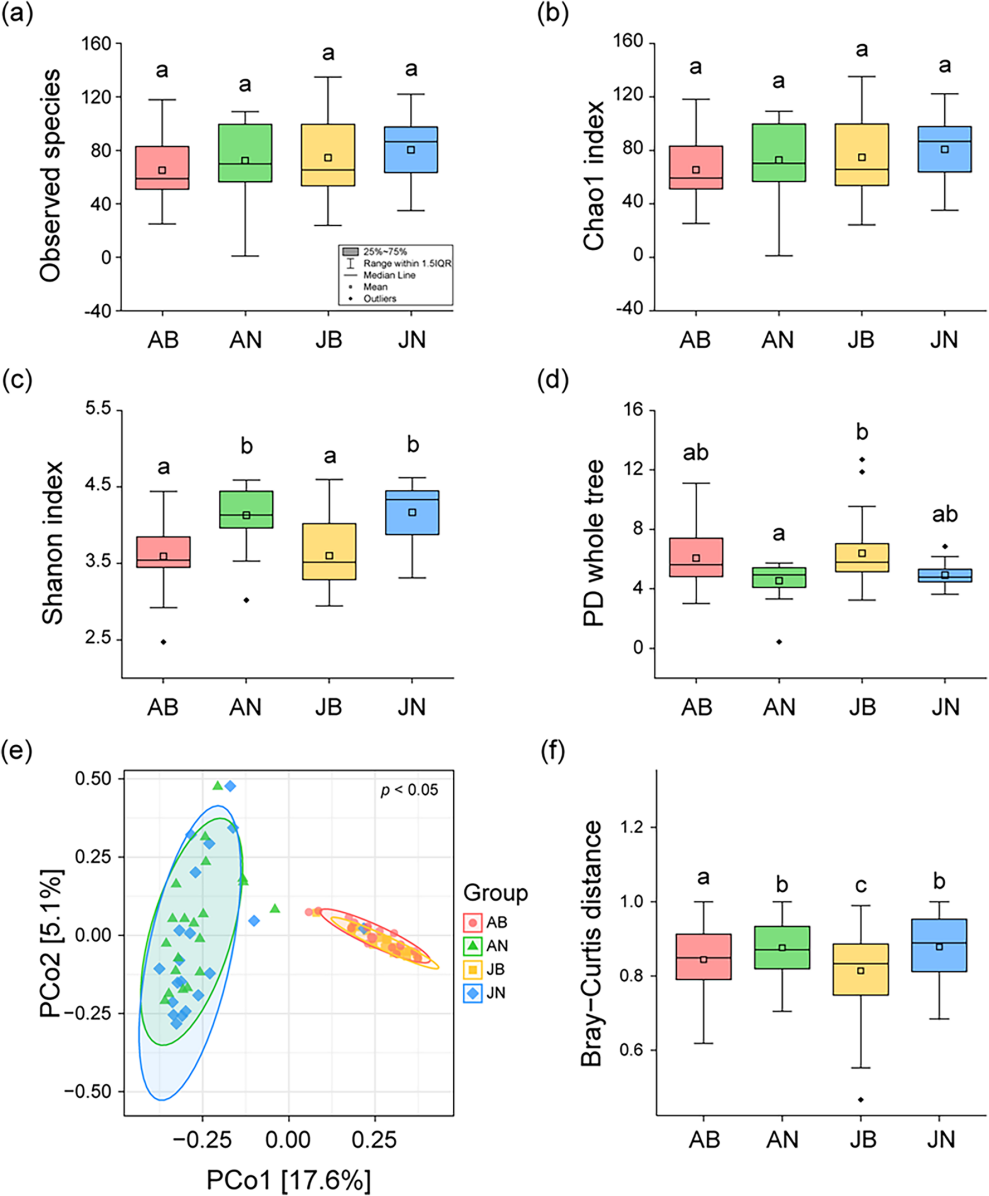
Alpha and beta diversity of gut bacterial communities of frogs by season and life history: (**a**) Observed species, (**b**) Chao1 index, (**c**) Shannon index, (**d**) phylogenetic diversity (PD) whole tree, (**e**) scatter plot of principal coordinate analysis (PCoA) based on Bray–Curtis dissimilarity, and (**f**) dissimilarities of gut bacterial communities among the four groups. Significant differences (*p* < 0.05) were determined using a Dunn’s post hoc test following a Kruskal–Wallis test, and are indicated by lowercase letters in panels (**a**), (**b**), (**c**), (**d**), and (**f**).

We compared the alpha diversity, and found that the number of observed species (H = 3.69, *p* = 0.30) and chao1 index (H = 3.69, *p* = 0.30) did not exhibit significant differences among the four groups (Fig. 2a–b). The Shannon index, based on species heterogeneity (H = 25.28, *p* < 0.01), and the PD (phylogenetic diversity) whole tree (H = 12.72, *p* < 0.01) showed significant differences among the four groups (Fig. 2c–d). In the comparison of the Shannon index, no significant difference was found between adult and juvenile frogs (Dunn's post hoc, *p* > 0.05); however, seasonal differences in diversity were observed. The Shannon index in the frogs from the non-breeding season was higher than that in the frogs from the breeding season (Dunn's post hoc, *p* < 0.05). In the comparison of the PD whole tree diversity, significant differences were observed only between adult frogs in the non-breeding season and juvenile frogs in the breeding season (Dunn's post hoc, *p* < 0.05), attributed to the combined effects of life history and seasonal variations.

Beta diversity also explains the strong difference of composition in gut bacterial community between breeding and non-breeding seasons, rather than differences between adult and juvenile frogs. PERMANOVA analysis revealed significant differences in the bacterial community composition among the four groups (R^2^ = 0.18, F = 5.71, *p* < 0.05). PCo1 and PCo2 contributed to only 22.7% of the variance (Fig. 2e); nonetheless, differences of composition in bacterial community among groups were significant (Bray–Curtis dissimilarity, *p* < 0.05). PCoA accounted for differences of composition in the bacterial community during the breeding season. In comparison with the Bray–Curtis dissimilarity, the composition of the bacterial community was significantly divided by the breeding season (Dunn's post hoc, *p* < 0.05). Furthermore, within the breeding season, there was a significant division in bacterial community composition between adult and juvenile frogs (Dunn's post hoc, p < 0.05) (Fig. 2f).

### Composition of bacterial community and functional group

**Figure 3:**
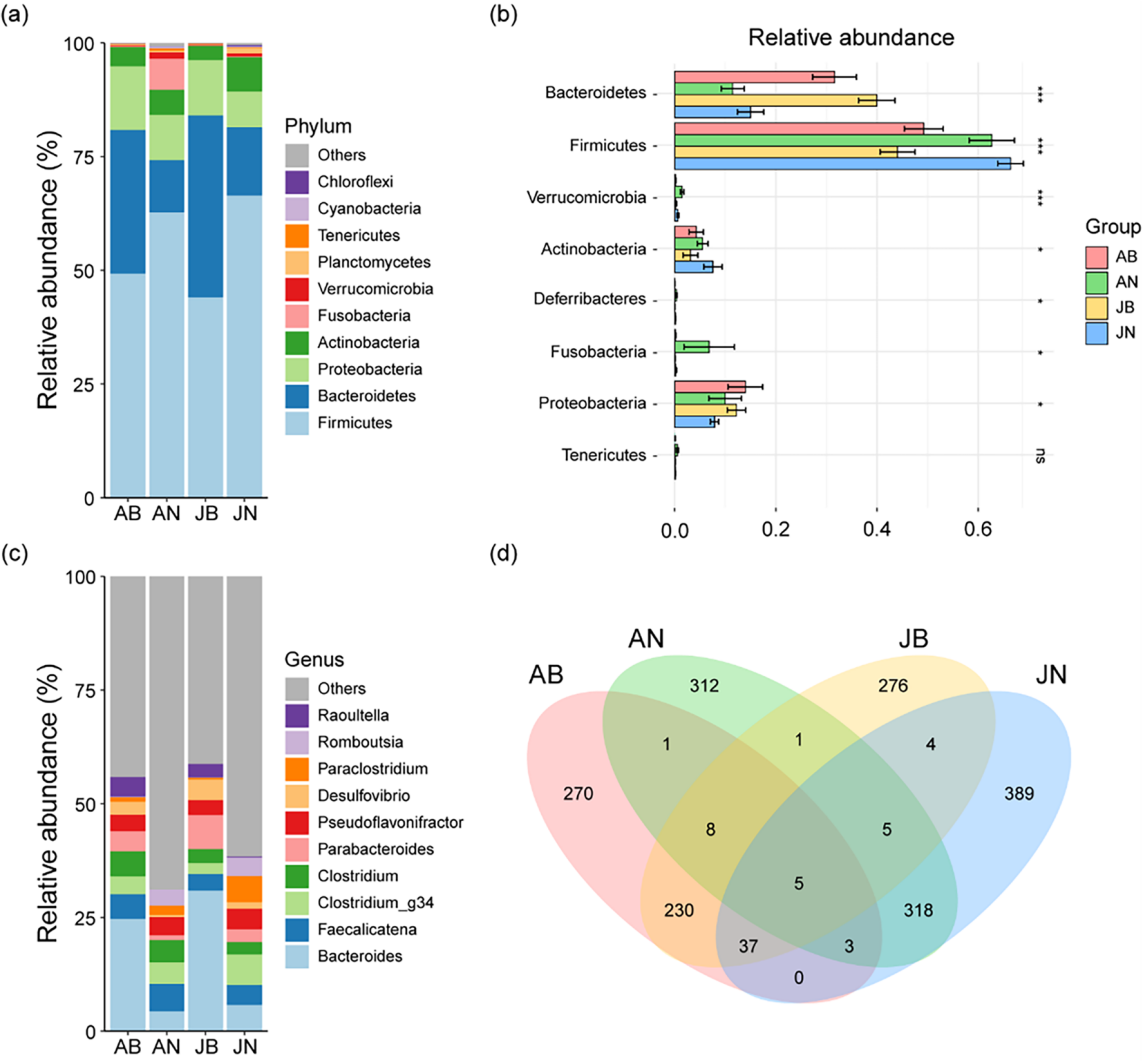
Relative abundance and Venn diagram illustrating the overlap of gut bacterial taxa in frogs, segmented by season and life history: (**a**) The composition of the bacterial community at the phylum level across the four groups, (**b**) A comparison of the relative abundance of bacterial taxa at the phylum level, where significant differences are marked with asterisks (* for *p* < 0.05, *** for *p* < 0.001) and non-significant differences are noted as 'ns'. Significant differences were identified using a Kruskal–Wallis test, (**c**) The composition of the bacterial community at the genus level across the four groups, (**d**) A Venn diagram depicting the overlap of gut bacterial operational taxonomic units (OTUs) among the four groups.

At the phylum and genus levels, the relative abundance of the gut bacterial composition significantly differed in frogs between the breeding season and the non-breeding season, although not between adult and juvenile frogs. At the phylum level, among the four groups, the gut bacterial communities of frogs were dominated by Firmicutes, Bacteroidetes, and Proteobacteria (Fig. 3a). In particular, Firmicutes (H = 24.416, *p* < 0.001) and Bacteroidetes (H = 32.434, *p* < 0.001) had remarkable differences in the four groups (Fig. 3b). The relative abundance of Firmicutes in frogs the breeding season (adult; 49.24%, juvenile; 44.05%) was lower (Dunn’s post hoc, *p* < 0.05) than that in the non-breeding season (adult; 62.72%, juvenile; 66.41%). Contrastingly, Bacteroidetes showed higher (Dunn’s post hoc, *p* < 0.05) relative abundances in frogs from the breeding season (adult; 31.59%, juvenile; 39.94%) than those from the non-breeding season (adult; 11.46%, juvenile; 15.01%). At the genus level, *Bacteroides* dominated most of the gut bacterial composition in the breeding season both adult (24.96%) and juvenile frogs (32.14%), whereas in the non-breeding season, *Faecalicatena*, *Bacteroides*, *Clostridium*, and *Pseudoflavonifractor* dominated evenly (Fig. 3c). In Venn diagram, there was less overlap of gut bacterial Operational Taxonomic Units (OTUs) in frogs from the breeding season and frogs from the non-breeding season than that in adult and juvenile frogs, resulting in clear differences in composition of the gut bacterial community in seasons (Fig. 3d). The number of unique OTUs was 270 in adult frogs during the breeding season, 312 in adult frogs during the non-breeding season, 276 in juvenile frogs during the breeding season, and 389 in juvenile frogs during the non-breeding season. The overlapping OTUs in adult and juvenile frogs from the breeding and non-breeding seasons were 17 and 51, respectively. Contrastingly, the overlapping OTUs of adult and juvenile frogs in the breeding and non-breeding seasons were very high at 280 and 331, respectively.

**Figure 4:**
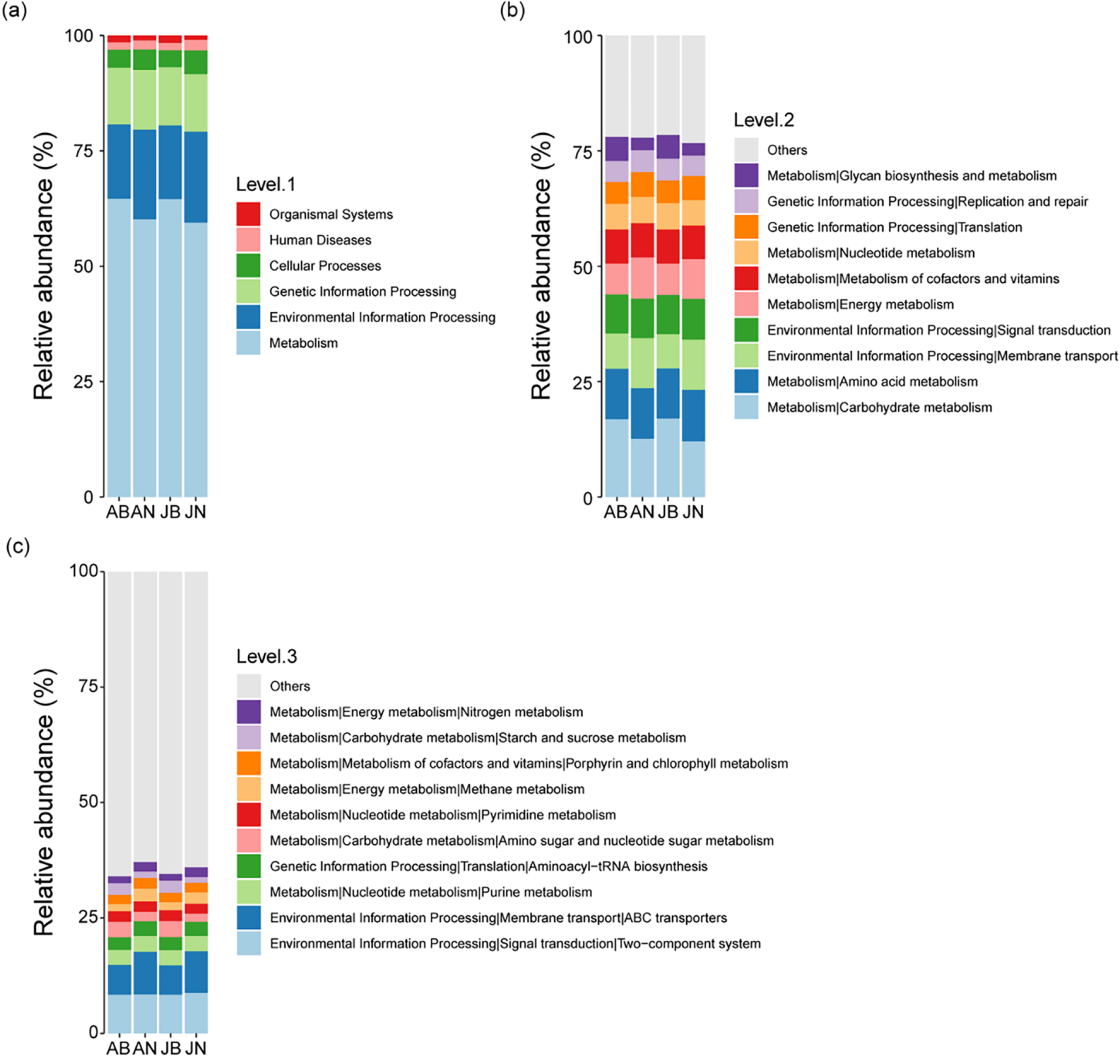
Relative abundance of annotated functional groups of gut bacterial communities of frogs by season and life history: (**a**) Top level of relative abundance in functional groups, (**b**) second level of relative abundance in functional groups, and (**c**) third level of relative abundance in functional groups.

The abundance of functional groups in the gut bacterial composition also differed by the breeding season. Metabolism was the function with the highest relative abundance in the four groups, and environmental and genetic information processing were the bacterial functional groups with the next highest relative abundance (Fig. 4a). During the breeding season, both adult and juvenile frogs exhibited a higher relative abundance of functional groups related to metabolism and a lower relative abundance of functional groups involved in environmental information processing compared to their counterparts in the non-breeding season. At the second enrichment level, the relative abundance of metabolism in functional groups was mainly related to carbohydrate and amino acid metabolism, and energy metabolism. During the breeding season, frogs exhibited relatively high carbohydrate metabolism, but low amino acid and energy metabolisms. The relative abundance of functional groups involved in environmental information processing, specifically membrane transport and signal transduction, was found to be lower during the breeding season compared to the non-breeding season (Fig. 4b). At the third enrichment level, the functional groups of ABC transporters, amino sugar and nucleotide sugar metabolism, methane metabolism, and starch and sucrose metabolism were those with prominent differences. During the breeding season, frogs exhibited a lower relative abundance of ABC transporters and methane metabolism, and a higher relative abundance of amino sugar and nucleotide sugar metabolism (Fig. 4c).

### Difference of immunity and body composition

Body length and weight did not significantly differ between seasons for adult frogs (Dunn's post hoc, *p* > 0.05). In contrast, juvenile frogs exhibited significantly lower body length and weight during the breeding season compared to the non-breeding season (Dunn's post hoc, *p* < 0.05) (Table 1). Bacterial killing ability (BKA), which represents the innate immunity of complement systems, fat content ratio, and lean body content ratio, had the significant difference estimated by combination in life history and seasonal difference, among the four groups (Table 1). Juvenile frogs in breeding season had a lower BKA than that of the other groups, and only adult frogs in the non-breeding period had a higher fat content and lower lean body mass than that of the other groups.

**Table 1:** Mean ± standard deviation (SD) and comparison of body length, body weight, and physiological conditions (bacterial killing ability; BKA, fat content ratio, and lean body content ratio) of adult breeding (AB), adult non-breeding (AN), juvenile breeding (JB), and juvenile non-breeding frogs (JN). Significant differences (*p* < 0.05) were determined using a Dunn’s post hoc test following a Kruskal–Wallis test, and are indicated by lowercase letters.

Contents	Adult		Juvenile	
	Breeding	Non-breeding	Breeding	Non-breeding
SVL (mm)	64.28 ± 4.5^a^	66.87 ± 4.11^a^	41.71 ± 6.95^b^	55.31 ± 3.3^c^
Mass (g)	22 ± 4.67^a^	24.36 ± 4^a^	6.64 ± 1.68^b^	11.67 ± 1.26^c^
BKA (%)	99.08 ± 0.83^a^	99.3 ± 1.21^a^	71.76 ± 22.89^b^	98.17 ± 1.83^a^
Fat (%)	15.64 ± 2.54^a^	19.26 ± 2.07^b^	16.5 ± 3.73^a^	16.1 ± 3.8^a^
Lean (%)	80.78 ± 2.47^a^	77.34 ± 1.77^b^	81.12 ± 3.48^a^	81.14 ± 3.41^a^

### Relationship of food resource, gut bacterial community, and physiological conditions

**Figure 5:**
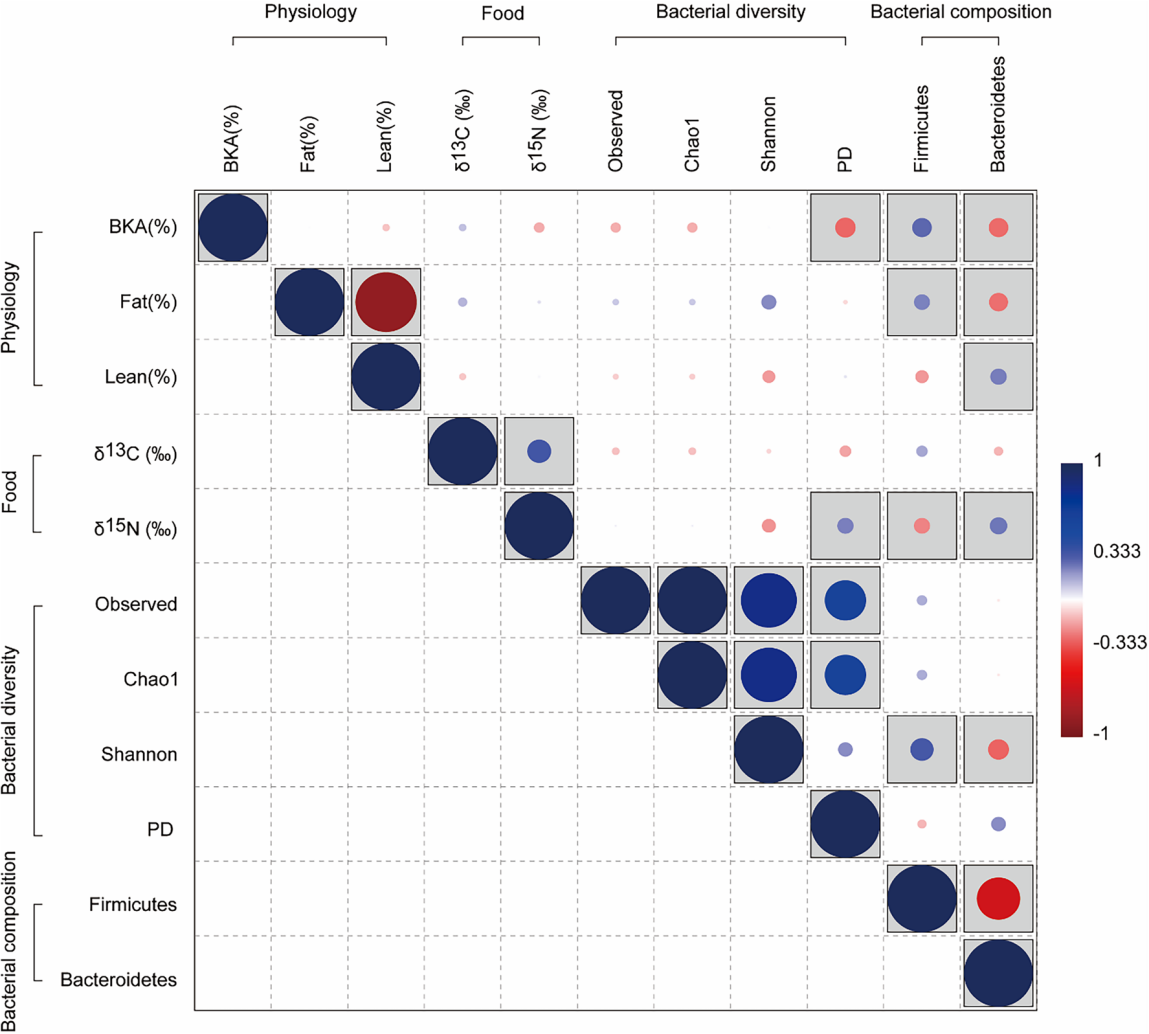
Correlation among food sources (δ^13^C and δ^15^N), gut bacterial diversity (observed species, Chao1 index, Shannon index, and phylogenic diversity whole tree; PD), relative abundance of the two dominant bacterial phyla in the gut (Firmicutes and Bacteroidetes), and physiological condition (bacterial killing ability; BKA, fat content, and lean body content) by Spearman correlation analysis. The color gradient of the correlation plot indicates the Spearman correlation coefficient, and gray boxes indicate significant correlations (*p* < 0.05).

Spearman's correlation analysis of the food source contents, gut bacterial diversity, relative abundance of Firmicutes and Bacteroidetes, and physiological conditions showed a correlation among some contents (Fig. 5). Observed species, Chao1 index, and Shannon index, which are metrics of alpha diversity, did not show significant correlations with food sources or physiological conditions (*p* > 0.05). A significant correlation (Spearman's R = 0.35, *p* < 0.05) between δ^13^C and δ^15^N was observed, and particularly, δ^15^N significantly correlated with the PD whole tree (Spearman's R = 0.24, *p* = 0.04) and the relative abundance of Firmicutes (Spearman's R = − 0.23, *p* = 0.04) and Bacteroidetes (Spearman's R = 0.25, *p* = 0.02), explaining the relationship between food source and gut bacterial community. However, δ^15^N had no direct correlation (*p* > 0.05) with items representing the physiological state. Contrastingly, the PD whole tree and relative abundance of Firmicutes and Bacteroidetes significantly correlated with physiological conditions. Increases in BKA correlated with decreases in PD whole tree (Spearman's R = − 0.30, *p* = 0.01), increased relative abundance of Firmicutes (Spearman's R = 0.28, *p* = 0.01), and decreased relative abundance of Bacteroidetes (Spearman's R = − 0.28, *p* = 0.01). An increase in fat content explained an increase in the relative abundance of Firmicutes (Spearman's R = 0.23, *p* = 0.04) and a decrease in the relative abundance of Bacteroidetes (Spearman's R = − 0.27, *p* = 0.01), whereas an increase in lean body content explained an increase in the relative abundance of Bacteroidetes (Spearman's R = 0.23, *p* = 0.03).

## Discussion

### Phenological difference and relationship of food source and gut microbiota

In this study, we confirmed that differences in food sources, gut bacterial community, and host physiology are influenced by the combined effects of seasonal changes and life history. Seasonality significantly contributed to variations in food sources and the diversity and composition of the gut bacterial community. The physiology of the host appeared to reflect the combined influences of seasonal differences and life history. The gut bacterial community was particularly closely related to host physiology, with correlations also observed with certain food source components. However, no direct correlation was identified between host physiology and food sources.

The differences in δ^15^N and SEAc values, representing the trophic level and food availability, respectively, revealed seasonal difference. Particularly, δ^15^N was associated with certain aspects of gut bacterial diversity and composition, but not immunity or body composition. Generally, frogs are opportunistic predators, and their food sources are often determined by body size (including head size) and environmental resource availability^37,38^. However, adult frogs allocate less energy to somatic growth due to reproductive efforts; thus, the reduced food availability during the non-breeding season, as compared to the breeding season, may stem from seasonal variations in environmental resources rather than differences in body size. This is expected as the composition of prey insects in temperate regions greatly varies with the seasons^39^. Our findings suggest a combination of growth and seasonality determines the differences in trophic level and food availability in juvenile frogs. Unlike adults, juveniles allocate energy to somatic growth year-round instead of focusing on breeding.

We also observed correlations between the relative abundances of Firmicutes and Bacteroidetes and δ^15^N, including PD. This is likely because seasonal differences in food source and gut bacterial community strengthened the relationship between the two variables. The patterns of differences in certain indices of gut bacterial diversity mirrored the differences in δ^15^N. Differences in gut microbiota according to food resource availability, confirmed by gut contents according to seasonal changes, were reported earlier^23^. However, akin to our study, differences in food sources were ultimately incorporated into seasonal changes. As a result, the combined effects of seasons and life history can amplify this relationship. While food sources undoubtedly affect changes in the gut microbiome, we suggest that seasonality may have a stronger effect in ectothermic animals. To gain a better understanding, the strength of the direct correlation between food sources and gut microbiota needs to be validated through laboratory experiments.

However, physiological parameters did not show any correlation with food conditions. Therefore, we postulate that instead of a direct correlation between physiological parameters and food sources, the gut bacterial community could mediate the response between these two factors. To elucidate more detailed processes and clarify their interrelationships, future studies will need to explore manipulations of food sources under laboratory conditions.

### Relationship of gut microbiome and host physiology strengthen by combined effect of seasons and life history

Seasonal differences strongly influence variation in gut bacterial diversity and composition, more so than life history. Some comparisons revealed the result of a combined effect of seasonal differences and life history. The PD whole tree showed statistical differences between adult frogs from the non-breeding season and juvenile frogs from the breeding season. Contrastingly, prior research has indicated that there is no significant gap in the diversity and composition of gut microbiota between adult and juvenile frogs^8,40^. These studies seemingly contradict the seasonal patterns of gut microbiota found in both endothermic and ectothermic animals^41,42^. For endothermic animals, the story is more complex. Changes in microbiota according to external temperature arise from metabolic alterations, all aimed at maintaining body temperature^43^. Ectothermic animals, on the other hand, see most alterations in their microbial community due to changes in body temperature itself, along with related physiological factors^44^. Gut microbial diversity may provide physiological stability by modulating resistance or resilience in different environments that hosts and populations may experience^45^. Our results support the fact that gut bacterial diversity strongly varies with seasonal change and additionally suggest that differences can be enhanced when combined with life history.

The ecological context shaped by combined effect of seasons and life history seems to significantly impact the variations of the bacterial phyla Firmicutes and Bacteroidetes. Firmicutes play a crucial role in carbohydrate fermentation and enhancing nutrient uptake efficiency^46^. In addition, the symbiotic relationship between Firmicutes and Bacteroidetes is notable, with the ratio of these two microbial populations being associated with conditions like obesity, characterized by high fat mass^47^. A high proportion of Firmicutes is likely to foster fat absorption and energy storage in the host. This process effectively boosts the efficiency of caloric intake^48,49^. In the non-breeding season, the relative abundance of Firmicutes surpassed that of the breeding season. This trend aligns well with the primary activities during the non-breeding season for frogs: feeding, digestion, and energy storage in anticipation of hibernation. Our observations further indicate that functional groups related to digestion predominantly exist in both adult and juvenile frogs during the non-breeding season. Typically, fat content in frogs tends to peak just before hibernation, in the period between summer and fall, preparing them for successful hibernation and the subsequent breeding season^17,50^. This relationship also appears to be linked to the increased prevalence of the genus *Clostridium*, which could be important for energy absorption during the non-breeding season^51^. The observed correlation between higher levels of Firmicutes and increased fat content suggests that this bacterial group may support fat storage at this time. This likely occurs through a close interaction with the host's physiology, illustrating a complex and fascinating interplay between the gut microbiota and the host's metabolic processes.

Members of the Bacteroidetes phylum aid in immune and intestinal microbalance by degrading polysaccharides, facilitating nutrient utilization, developing the immune system, and assisting in intestinal mucosal vascularization^52^. The high relative abundance of Bacteroidetes in the breeding season may play different physiological support roles in adult and juvenile frogs. Decreases in immunity were found only in juvenile frogs during the breeding season, while lower fat content and higher lean body content were observed exclusively in adult frogs during the non-breeding season. After hibernation, adult and juvenile frogs appear in paddy fields for breeding and growth, respectively^12,53^. Adult frogs experience explosive energy consumption for reproduction during the breeding season, and juvenile frogs must recover their immunity in a decreased immune state. Unlike juvenile frogs, adult frogs exhibit reproductive behavior rather than growth during the breeding season, showing a low fat content during the breeding season among the annual rhythms^50^. In the case of male frogs, energy consumption is considerable during the breeding season because they make advertisement calls, move to find females, and find and protect territories favorable for breeding^54^. This energy usually comes from glucose and stored fat^55^, and the abundance of Bacteroidetes in adult frogs during the breeding season suggests that this metabolism may be bacterial assisted. In fact, the functional groups related to glucose metabolism were dominant in adult frogs during the breeding season. Similar patterns are observed at the genus level. The genus *Bacteroides*, which increases during the breeding season, plays a role in sensing nutrient availability and facilitating energy utilization. The observed decrease in fat content alongside an increase in *Bacteroides*, a member of the phylum *Bacteroidetes*, may reflect a relationship strengthened by both seasonal changes and life history. This suggests that the gut microbiota, particularly *Bacteroides*, may support the reproductive activities of adult male frogs by aiding in metabolic processes.

Immunity can be lowered during hibernation or at low temperatures because the low temperatures increase the energy costs of immune cell production^53^. Decreased immunity requires time for recovery^53^. In our study, this was only found in juvenile frogs. As expected, adults appeared to have relatively high immunity stability with minimal seasonal fluctuations. However, juvenile frogs possess a more immature immune status compared to adult frogs; thus, the decreased immunity observed in juvenile frogs during winter appears to recover more slowly as the spring season progresses than in adult frogs. In this process, Bacteroidetes, which play a role in developing the immune system, may exist in a high state to support immune recovery in juvenile frogs during the breeding season^52^. Within the Bacteroidetes phylum, *Bacteroides* was notably abundant during the breeding season and is also associated with the host immune system and pathogen control^56^. The contrast in immune states and the high levels of *Bacteroides* in juvenile frogs between breeding and non-breeding seasons illustrate this association. The observed negative correlations between these two factors underscore a relationship strengthened by phenological changes. Furthermore, the distinct physiological responses observed in adult and juvenile frogs indicate that similar bacterial community shifts within the same season can support different physiological outcomes depending on the amphibian's life history.

From a physiological viewpoint, BKA may also be influenced by energy trade-offs^57,58^, suggesting that frogs with high fat, exhibiting a high-energy state, may also have increased innate immunity. Immune balance is influenced by several steroid hormones, notably those involved in energy metabolism and reproduction. An increase in corticosterone, which responds to stress, may reduce immunity^59^, while testosterone supplementation often boosts it in larger males^60^. Moreover, this regulation of physiological immunity is affected not just by a range of environmental factors but also by dietary control^61^. Additionally, in frogs, high fat content is positively associated with fertility, influencing the fertilization success in male frogs as well as egg and clutch sizes in female frogs^62,63^. High fat contents, characterized by a narrow prey spectrum and low activity in captive frogs, is considered detrimental for individual frogs^64^, but this does not seem to be the case in wild populations. We also demonstrated the frogs most vulnerable to disease or those with weak immune responses are likely to be juvenile frogs from winter to spring. Since the increased immunity of juvenile frogs during the non-breeding season reaches the same level as in adult frogs, a frog can be comparatively safe once it has passed through the spring season.

## Conclusion

**Figure 6:**
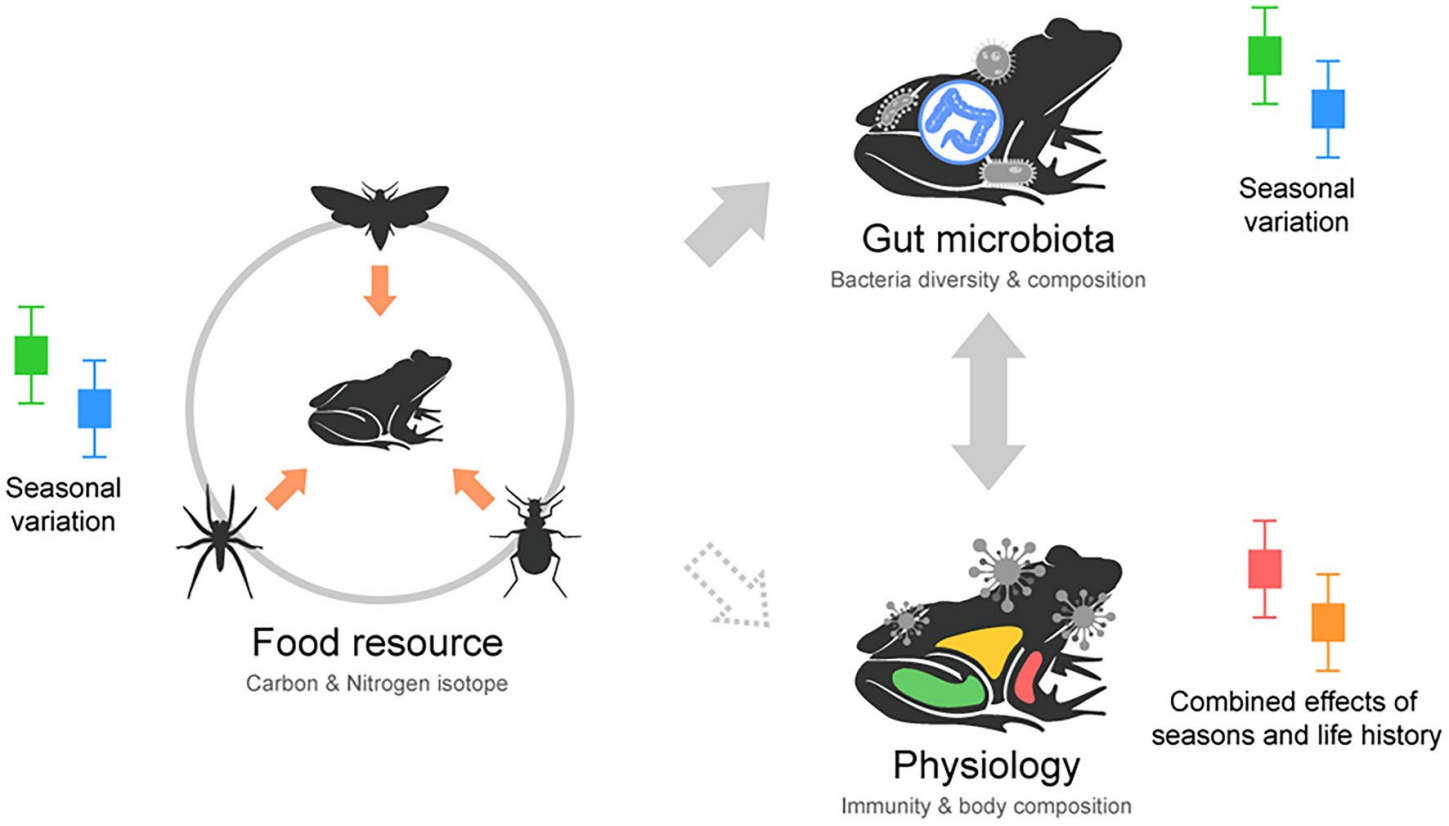
A diagram for the conclusion of this study. Gray arrows indicate direct correlations, while dashed arrows indicate potential relationships that may be indirect. Seasonal variation cause to be strong differences in the three traits, and combined effect seasonal variation and life history strengthened the relationship between physiology and the gut bacterial community. Additionally, the gut bacterial community can serve as a bridge connecting the relationship between food source and physiology.

Seasonal differences mainly influenced food sources, gut bacterial diversity, and host physiology. Phenological elements like life history and seasonal shifts appear to exert a more profound impact on gut microbiome changes, relegating food sources to a lesser role (Fig. 6). In the end, seasonal factors, including temperature, heavily guide ectothermic animals, and their responses can be magnified with life history considerations.

While innate immunity had high volatility in juvenile frogs, changes in fat content hinged solely on reproductive metabolism of adult frogs. Conversely, variations in gut microbiota, including aspects like bacterial diversity and composition, were consistent across life stages. We thus infer that juvenile frog may not be more susceptible to gut bacterial community shifts during seasonal transitions, such as from spring to summer, unlike immunity. However, since this conclusion may specifically apply to the species studied, future research across multiple species and taxa is necessary. Also, extreme conditions like hibernation might tell a different story.

Importantly, our research stands out in identifying a direct link between gut microbiota and the host's physiological state in ectotherms. Moreover, the correlations found between food sources and host physiology hint at the gut microbiota serving as an intermediary connection. These findings enrich our understanding of microbial communities and complex physiological responses in ectothermic animals. Still, the intricate nature of physiological processes, governed by various intertwined factors, calls for a more thorough investigation. The existing insights shed light on this multifaceted relationship but also highlight the need for continued exploration.

## Methods and materials

### Field investigation

Black-spotted pond frogs (*P. nigromaculatus*) are known to be highly philopatric, meaning they often remain in the same location throughout both breeding and non-breeding seasons, engaging in breeding and feeding behaviors^65^. Juvenile frogs, which do not reproduce, also appear at breeding sites after wintering and immediately begin feeding.

**Figure 7:**
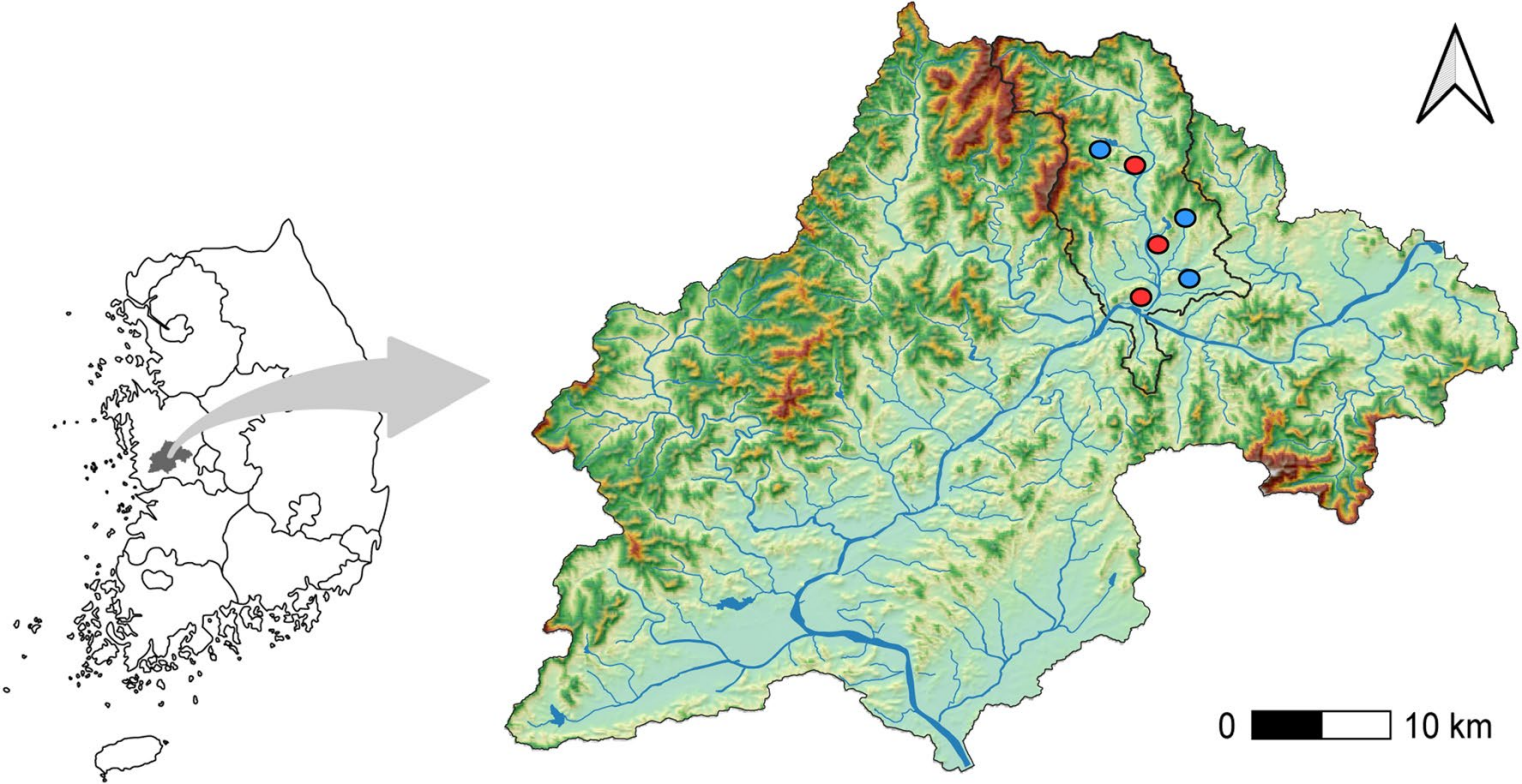
Black-spotted pond frogs (Pelophylax nigromaculatus) sampling sites. This map illustrates six sampling sites for Black-spotted Pond Frogs. Three sites are located in plain paddy fields and three in terraced paddy fields, all indicated by red circles. The map was generated using QGIS software (version 3.28.0), available at https://qgis.org.

We collected both adult and juvenile frogs in May 2022, during the breeding season, from six paddy fields in Gongju City (Fig. 7). All adult frogs collected were males, identified by the presence of a vocal sac and nuptial pads on the first finger, which are secondary sexual characteristics. Juvenile frogs were distinguished as individuals with a smaller body size lacking male characteristics. In the field, individuals that did not clearly fit these distinctions were not collected. To minimize the potential effects of regional characteristics, topography, and cultivation practices, we selected three plain paddy fields and three terraced paddy fields for study. Although the water composition and altitude of these two types of paddy fields are similar, plain paddy fields are larger and accommodate mechanized farming. In contrast, terraced paddy fields have not been subject to land consolidation and are located on sloping hillsides, surrounded by forests. From each site, three to four frogs were collected, resulting in a total of 20 adult and 20 juvenile frogs collected during the breeding season. The same six sites were used to collect adult and juvenile frogs in August 2022, during the non-breeding season, with the same number of individuals per group, three to four adult and juvenile frogs per site. The study's total sample comprised 80 individuals: 20 adult breeding (AB), 20 adult non-breeding (AN), 20 juvenile breeding (JB), and 20 juvenile non-breeding frogs (JN).

Frogs were immediately transported to the Kongju University Animal Laboratory within one hour of collection on the day of capture. All experimental procedures involving animals were conducted in accordance with the regulations and with the approval of the Experimental Animal Ethics Committee of Kongju National University (KNU_2022-01).

### Blood extraction and immune assay

In response to acute stress, the bacterial killing ability (BKA), which represents innate immunity, may change within 15 h^66,67^. Therefore, blood from the frogs was extracted within 2 h of collection. To alleviate the pain from the syringe needle, we anesthetize the frogs using a 0.5 g/L MS-222 (tricaine methane sulfonate) solution. This was buffered with sodium bicarbonate, referring to the concentrations and methods found in previous studies^68,69^.

The procedure was precise: After laying down the frog to check for the loss of the righting reflex, we used a wet cotton swab to confirm the loss of the corneal reflex. Then, blood was drawn from the fully anesthetized individual through cardiac venipuncture with a sterile syringe. Blood samples were swiftly transferred to heparinized vacuum tubes (SST) and spun at 3000 × g for 10 min. We collected the supernatant to obtain a plasma sample for the bacterial killing assay, storing it at − 40 °C until analysis. Samples stored at − 40 °C were analyzed within 1–2 weeks. After completing the blood extraction, the snout-vent length was measured with digital calipers, and body mass was recorded using a digital balance. Next, we performed bacterial killing assays, adhering to protocols from an earlier study^70^. We started by diluting the plasma sample 20-fold with amphibian Ringer's solution. Then, we added a working solution of non-pathogenic *Escherichia coli* (Microbio-Logics #24 311-ATCC 8739, St Cloud, MN, USA) to all samples. Carefully prepared controls included both positive and negative configurations. Everything was incubated at 37 °C for an exact hour. After this incubation, 500 µL of tryptic soy broth (TSB) was carefully added to both the samples and controls using a pipette. The mixed bacterial suspension was then transferred to a 96-well microplate in duplicate. After a 2-h incubation, we embarked on a meticulous process of measuring the bacterial optical density, using a microplate spectrophotometer at a 600 nm wavelength, taking six readings at 1-h intervals. By taking six readings, we identified the growth phase of the bacteria, at which point the BKA was calculated. Finally, BKA was calculated according to a specific formula [1 − (optical density of the sample/optical density of the positive control)], providing an indication of the proportion of killed bacteria in the plasma samples relative to the positive control.

### Analysis of gut bacterial community

To analyze the gut bacterial community of the frogs, each individual was euthanized by pithing after anesthesia prior to dissection. The dissection was performed immediately after measuring body length and weight. To prevent cross-contamination, frogs were dissected on a clean bench using sterilized equipment such as scissors and tweezers. The frog's peritoneum was opened, the starting point of the small intestine and end points of the large intestine were removed, and the sample was transferred to a tube containing beads provided by the Qiagen DNeasy PowerSoil Pro Kit (Qiagen, Hilden, Germany). Afterward, the tweezers and scissors were sequentially flame sterilized, alcohol sterilized, and washed with sterile water, and the intestinal samples were cut several times. Frogs from which the intestinal samples were extracted were stored in 99.5% ethanol for further analysis.

Extracting genomic DNA from the gut was done using the Qiagen DNeasy PowerSoil Pro Kit. After adding lysis buffer to the tube, we homogenized the sample using a TissueLyser II. The extraction process followed the manufacturer's instructions, and the genomic DNA was quantified using a Denovix-QFX fluorometer (DeNovix Inc., Wilmington, DE, USA) and a QFX dsDNA High Sensitivity Assay Kit. All samples were confirmed to have no abnormalities after electrophoresis was used to check the genomic DNA quality.

Bacterial genomic DNA extracted from frog intestinal samples was used to amplify the 16S ribosomal RNA gene (16S rRNA gene) region. Forward and reverse primer targeting the 16S rRNA gene V4 region containing the overhang adapter sequence provided by Illumina were used to amplify the target region: 515F: 5′-TCGTCGGCAGCGTCAGATGTGTATAAGAGACAGGTGCCAGCMGCCGCGGTAA-3′) and reverse primer (806R: 5′-GTCTCGTGGGCTCGGAGATGTGTATAAGAGACAGGGACTACHVGGGTWTCTAAT-3′. We conducted PCR under the conditions outlined in 'Preparing 16S Ribosomal RNA Gene Amplicons for the Illumina MiSeq System'^71^, utilizing the KAPA HiFi HotStart Ready Mix (Kapa Biosystems Inc., Wilmington, USA). After purifying the amplified PCR products with the Agencourt AMPure XP PCR purification system (Beckman Coulter, Brea, CA, USA), we conducted index PCR with the Nextera XT Index Kit (Illumina, San Diego, CA, USA). After secondary PCR, the products underwent another purification round using the same purification system. Concentration measurement of the purified index PCR product and electrophoresis confirmed the size and quality of the PCR products. The samples were pooled, diluted, and analyzed using an Illumina MiniSeq system (Illumina, San Diego, CA, USA).

Paired-end sequencing data in the FASTQ format were analyzed using QIIME2 (v.2022.2) and associated plugins. The DADA2 pipeline was employed for quality filtering, noise removal, merging denoised paired-end sequences, and feature-table construction. Trimming parameters were set based on Demux visualization. Taxonomic assignment of appropriate taxa was performed using the default settings of the feature-classifier plugin for QIIME2 against the EzBioCloud 16S reference database (https://www.ezbiocloud.net/, accessed on January 19, 2023). The dataset was filtered to contain only bacteria. The dataset was filtered to contain only bacteria. As a result, the dataset was classified into operational taxonomic units (OTUs) with 97% similarity. Functional group predictions were analyzed using the Tax4Fun functional classification database (http://tax4fun.gobics.de, accessed on July 23, 2023), which categorized OTUs based on sequence similarity^72^.

### Body composition measurements

After euthanizing the frogs, we removed all internal organs, including gastrointestinal tract, heart, lungs, liver, kidney etc. to focus our attention on the body's tissues from our study. The process required meticulous removal of everything from the esophagus' starting point to the cloacal cavity's end point, applying the same procedure to each individual. We then measured the body composition, including both fat and lean body content, utilizing dual-energy X-ray absorptiometry (DEXA; Medikors InAlyzer, Seongnam, Korea).

The DEXA analysis computes the lean body content as the sum of water and muscle content, a principle applied in our study^29,73^. We made efforts to remove the maximum body water by preserving tissue samples in 99.5% ethanol for a minimum of three months, ensuring consistency in the retention period across all samples. As a result, the lean body content we measured was seen as the muscle mass, free of most body water. The examination of fat and lean body contents went beyond mere analysis; it served as a tool to compare physiological conditions and discover the correlation between the gut bacterial community's composition and food sources.

### Stable isotope ratio

Following the measurements of body composition, we turned our attention to the hind limb muscles of the frogs, extracting them to explore the carbon (δ^13^C) and nitrogen (δ^15^N) stable isotope ratios. Since a previous study reported minimal changes in δ^13^C and δ^15^N for samples stored in ethanol for 8 weeks^74^, we considered our results to be stable as well. Transferred to 1.5 Ml microcentrifuge tubes, the muscles were lyophilized to rid them of moisture, then homogenized using a TissueLyser II (Qiagen, Hilden, Germany).

Lipid content can influence the carbon stable isotope ratio, as indicated by previously study^75^. To account for this, we performed a lipid removal procedure. The procedure involved the addition of a 2:1 (v/v) chloroform to 99.5% methanol mixture to the sample, followed by a 15-min sonication and centrifugation at 3000*g* for 5 min. This cycle was repeated three times, concluding with another round of lyophilization to remove residual solutions. However, for the nitrogen stable isotope ratio, we opted to use the homogenized samples without extracting lipids. This decision was guided by the risk of errors in stable isotope ratios when lipids are extracted^76^.

The subsequent step involved transferring 0.1–0.2 mg of the homogenized tissue samples into a 6 × 4 mm tin capsule using an electronic balance. Precision was key, so we used an electronic microbalance (ME204, Mettler Toledo, Japan) to ensure accuracy. The samples' stable isotope ratio was then analyzed with an isotope ratio mass spectrometer (EA-IRMS; EuroEA-Isoprime IRMS, GV Instruments, UK), employing the formula δX (‰) = ((R_sample/R_reference) − 1) × 1000, where "X" symbolizes either carbon or nitrogen, and "R" refers to the 12C/13C or 15N/14N ratio. We used atmospheric N2 as the standard reference material for nitrogen, and Vienna Pee Dee Belemnite (VPDB) for carbon.

### Statistical analysis

The availability of food resources was ascertained using the SIAR (Stable Isotope Analysis in R) package^77^. This was performed within a Bayesian mixing model, utilizing R software version 4.2.0 (https://www.r-project.org/)^78^. From this analysis, two key metrics were obtained: the standard ellipse area (SEA) to infer food resource availability, and a sample size-corrected standard ellipse area (SEAc), aimed at reducing SEA bias.

We employed the Kruskal–Wallis test to compare the differences in various parameters including SVL, body weight, BKA, Fat, Lean, δ^13^C, δ^15^N, and the alpha diversity of bacterial communities among four groups (AB, AN, JB, and JN). When a significant difference emerged from this test, we proceeded with Dunn's post hoc test. GraphPad Prism software, version 8.00 (GraphPad Software, San Diego, CA, USA), facilitated our statistical analyses.

Analyses of alpha and beta diversities in the bacterial communities were carried out with the *microeco* package^79^ in R software version 4.2.0 (https://www.r-project.org/)^78^. We scrutinized differences in alpha diversity indices (observed species, Chao1 index, Shannon diversity index, and phylogenetic diversity; PD), Bray–Curtis dissimilarity, and the relative abundance of gut bacterial taxa among four groups (AB, AN, JB, and JN) using the Kruskal–Wallis test. Dunn's post hoc test was employed for comparison when significant differences emerged.

A permutational multivariate analysis of variance (PERMANOVA) was utilized to analyze differences in bacterial community composition, and the results were visualized through principal coordinate analysis (PCoA). For bacterial taxonomic data, we carried out a Hellinger transformation and compared it with a dissimilarity matrix, which was constructed using the Bray–Curtis distance metric.

We conducted a comprehensive correlation analysis among various factors, including food sources (δ^13^C and δ^15^N), gut bacterial diversity metrics (observed species, Chao1 index, Shannon index, and PD whole tree), the relative abundances of Firmicutes and Bacteroidetes (the two dominant OTUs in the gut bacterial community), and physiological conditions such as BKA, fat content, and lean body content. The method of choice for this phase was Spearman correlation analysis, and we set the threshold for statistical significance at *p* < 0.05.

### ARRIVE guidelines

Animal experimental procedures and laboratory animal management were conducted with the approval of the Laboratory Animal Ethics Committee of the Kongju National University (KNU_2022-01). The study is performed in accordance with ARRIVE guidelines.

## Data Availability

The datasets used and/or analyzed during the current study available from the corresponding author on reasonable request.
